# Determining levels of linguistic deficit by applying cluster analysis to the aphasia quotient of Western Aphasia Battery in post-stroke aphasia

**DOI:** 10.1038/s41598-022-17997-0

**Published:** 2022-09-06

**Authors:** Zhijie Yan, Dongshuai Wei, Shuo Xu, Jingna Zhang, Chunsheng Yang, Xinyuan He, Chong Li, Yongli Zhang, Mengye Chen, Xiaofang Li, Jie Jia

**Affiliations:** 1 Department of Rehabilitation Medicine, The Third Affiliated Hospital of Xinxiang Medical University, The East Section of Hualan Avenue, Hongqi District, Xinxiang, 453000 China; 2 Department of Rehabilitation Medicine, Huashan Hospital, Fudan University, 12 Middle Wulumuqi Road, Jing’an District, Shanghai, 200040 China; 3 National Clinical Research Center for Aging and Medicine, Huashan Hospital, Fudan University, Shanghai, 200040 China

**Keywords:** Diseases, Neurology

## Abstract

The aphasia quotient of Western Aphasia Battery (WAB-AQ) has been used as an inclusion criterion and as an outcome measure in clinical, research, or community settings. The WAB-AQ is also commonly used to measure recovery. This study aimed to quantitatively determine levels of the linguistic deficit by using a cluster analysis of the WAB-AQ in post-stroke aphasia (PSA). 308 patients were extracted from the database. Cutoff scores are defined by mean overlap WAB-AQ scores of clusters by systematic cluster analysis, the method of which is the farthest neighbor element, and the metrics are square Euclidean distance and Pearson correlation, performed on the full sample of WAB-AQ individual subitem scores. A 1-way analysis of variance, with post hoc comparisons conducted, was used to determine whether clusters had significant differences. Three clusters were identified. The scores for severe, moderate, and mild linguistic deficit levels ranged from 0 to 30, 30.1 to 50.3, and 50.4 to 93.7, respectively. For PSA, the cluster analysis of WAB-AQ supports a 3-impairment level classification scheme.

## Introduction

The number of stroke individuals worldwide is still increasing year by year^1^. Aphasia after stroke accounts for about one-third of the total number of stroke survivors^2^. Stroke comprises a heterogeneous population with a wide range of linguistic deficits. To facilitate treatment planning and evaluation of progress in clinical, research, or community settings, stroke populations need to be comprehensively evaluated. Facing the increasing demand for stroke rehabilitation^3^, the Research Outcome Measurement in Aphasia (ROMA) consensus statement suggested WAB-Revised as the measurement of clinical outcomes^4^, and the WAB of the linguistic deficit is the most widely used assessment to measure PSA in the research context^5,6^. The WAB-AQ score has been used as an inclusion criterion and as an outcome measure for clinical trials^7,8^. To determine the optimal method to evaluate poststroke linguistic deficit, previous articles^6,9^ studied related assessment tools, including the use of the WAB.

The WAB has four subdomains^10,11^: (1) spontaneous speech; (2) auditory comprehension; (3) repetition; and (4) naming designed to determine the presence of aphasia and judge the type of linguistic deficit and measure the severity of language impairment. The full score of spontaneous speech is 20, including fluency and content of information. The 200 points of auditory comprehension consist of 60 points of Yes–No questions, 60 points of auditory word recognition, and 80 points of sequential commands. The total score of repetition is 100. A total of 100 points are given for naming, including 60 points for object naming, 20 points for fluency of words, 10 points for sentence completion, and 10 points for responsive speech. The calculation formula of the aphasia quotient is “AQ = (Spontaneous + Comprehension ÷ 20 + Repetition ÷ 10 + Naming ÷ 10) × 2”. All of the items result in a range of possible scores from 0 to 100. Combined with the clinical data of stroke patients, those whose aphasia quotient is less than 93.8 can be judged as aphasia, and the smaller the aphasia quotient, the more serious the aphasia^10^. In addition, the WAB-AQ is commonly used to measure recovery^12^, although there is a discrepancy between clinical impression and WAB in the classification of aphasia, the superiority of WAB is that it can quantify language damage^13^.

The current clinical reality is to use WAB-AQ to determine whether aphasia and its type. Compared with other common language clinical assessments, such as the Boston Diagnostic Aphasia Examination (BDAE), WAB-AQ has the advantages of the simple but quantifiable scoring system and relatively short execution time (approximately 1 h). Some studies^11,14^ have shown that the information content has a significant correlation with WAB-AQ scores in all sub-tests. We believe that the ratings of the individual WAB-AQ elements convey information that is lost when one considers only the WAB-AQ total score. The primary aims of our study were to use WAB-AQ individual items scores to (1) derive data-driven cutoff scores defining distinct levels of linguistic deficit; (2) determine the commonalities and differences of linguistic deficit within and between the severity levels.

## Methods

The data used in this report were obtained through our retrospective screening of the baseline evaluation database during the course of 2 studies of PSA conducted between 2020 and 2022^15,16^. The 2 studies used identical methodology for the collection of WAB-AQ data. A single speech therapist trained all 3 staff in the administration of the WAB-AQ. This study, which was conducted by relevant guidelines and regulations, was performed in line with the principles of the Declaration of Helsinki. Approval was granted by the Ethics Committee of Huashan Hospital, Fudan University. Patient consent was waived due to the retrospective nature of the study and the lack of patient interaction.

### Statistical methods

We performed cluster analysis with SPSS 25.0 (IBM Corporation, Armonk, NY, USA). In the analyses, all sub-test items of WAB-AQ including spontaneous speech, auditory comprehension, repetition, and naming were the independent variables. The method of systematic cluster analysis is the farthest neighbor element, and the metrics are square Euclidean distance and Pearson correlation. The optimal number of clusters was determined by selecting the largest, most discrete change in squared Euclidean distance or the minimum correlation coefficient between the adjacent number of clusters. A 1-way analysis of variance, with post hoc comparisons conducted using the Tamhane T2 Test, was used to determine whether clusters had significantly different mean WAB-AQ scores.

WAB-AQ cutoff scores defining the optimal clusters were identified by using the following methods. The participants were ranked according to the WAB-AQ total score from high to low, and each cluster member was checked. When cluster overlap occurred, the first one in the overlapping area was defined as high score, and the last one was defined as low score. The cutoff score was defined as the mean of the high score and low score^17^. This process was repeated to determine the cutoff scores between each adjacent cluster, thereby defining groups with similar severity of linguistic deficit.

The characteristics of linguistic deficit within each cluster were defined by the aggregate scores of the WAB-AQ sub-tests. The mean subtest scores of each group were compared between groups.

### Ethics statement

Approval was granted by the Ethics Committee of Huashan Hospital, Fudan University.

## Results

### Characteristics of participants

Our study included 308 individuals with PSA (200 male, 108 female; 101 Broca, 73 Global, 69 Anomic, 16 Wernicke, 16 Conduction, 15 transcortical motor, 12 Isolation, 6 transcortical sensory). The participants’ mean age was 60.5 ± 12.905 years, mean years of education was 10.89 ± 3.690, mean time post-onset (TPO) was 7.56 ± 9.629 months, and mean WAB-AQ score was 44.309 ± 29.997 (Table 1).

**Table 1:** Baseline characteristics of participants.

Items	
Number of individuals	308
**Gender**	
Female, *n* (%)	108 (35.1%)
Male, *n* (%)	200 (64.9%)
Age, years, mean ± SD (range)	60.5 ± 12.905 (18–89)
Time post-onset, months, mean ± SD (range)	7.56 ± 9.629 (0–42)
Education, years, mean ± SD (range)	10.89 ± 3.690 (0–18)
WAB-AQ scores, mean ± SD (range)	44.309 ± 29.997 (0–93.7)
**Types of aphasia**	
Broca’s aphasia, *n* (%)	101 (32.8%)
Global aphasia, *n* (%)	73 (23.7%)
Anomic aphasia, *n* (%)	69 (22.4%)
Wernicke’s aphasia, *n* (%)	16 (5.2%)
Conduction aphasia, *n* (%)	16 (5.2%)
Transcortical motor aphasia, *n* (%)	15(4.9%)
Isolation aphasia, *n* (%)	12 (3.9%)
Transcortical sensory aphasia, *n* (%)	6 (1.9%)

*SD* standard deviation, *WAB-AQ* aphasia quotient of Western Aphasia Battery.

### Cluster analysis and range of WAB-AQ scores within each cluster

**Figure 1:**
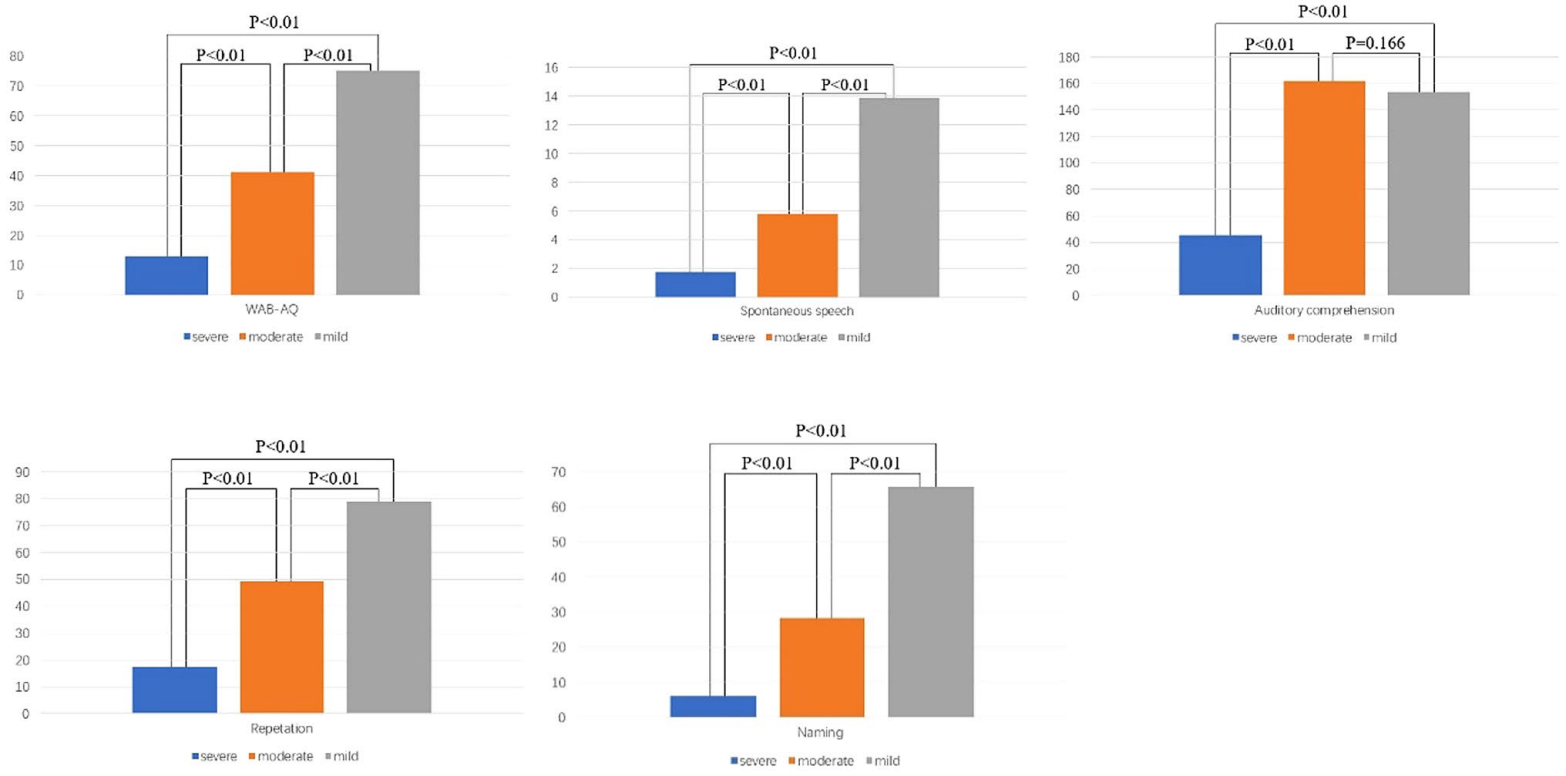
The characteristics of linguistic deficit within each cluster were defined by the aggregate scores of the WAB-AQ sub-tests. The mean subtest scores of each group were compared between groups.

The analysis identified 3 clusters. The number of participants in the three clusters is 109, 71, and 128 respectively. The mean score of WAB-AQ in the three clusters is 13.100 ± 12.001, 42.271 ± 16.918, and 72.017 ± 16.819 respectively. The mean months of TPO in the three cluster is 6.798 ± 9.746, 8.585 ± 9.899, and 7.408 ± 9.398 respectively. The results of univariate ANOVA showed that there was no significant difference in the mean TPO across the three clusters (p = 0.477). After selecting cutoff scores by using the method of mean overlap score described above, the ranges of scores for the 3 clusters were 0 to 30, 30.1 to 50.3, and 50.4 to 93.7. The WAB-AQ scores within the 3 clusters correspond to severe, moderate, and mild impairment levels. There were 46 aphasia patients within one month after the onset, including 20 severe aphasia, 6 moderate aphasia, and 20 mild aphasia. There were 154 patients with aphasia from 1 to 6 months after onset, including 62 patients with severe aphasia, 24 patients with moderate aphasia, and 68 patients with mild aphasia. There were 108 aphasia patients more than 6 months after onset, of which 41 were severe, 25 were moderate and 42 were mild. In the severe group, the mean scores of WAB-AQ, spontaneous speech, auditory comprehension, repetition, and naming were 13.058, 1.77, 45.61, 17.37, and 6.3. In the moderate group, the mean scores of WAB-AQ, spontaneous speech, auditory comprehension, repetition, and naming were 41.255, 5.79, 161.96, 49.21, and 28.37. In the mild group, the mean scores of WAB-AQ, spontaneous speech, auditory comprehension, repetition and naming were 75.235,13.9, 153.51, 78.,8 and 65.66. The results of ANOVA showed that the scores of WAB-AQ (p < 0.01), spontaneous speech (p < 0.01), repetition (p < 0.01), and naming (p < 0.01) were significantly different between groups, while there was no significant statistical difference in the scores of auditory comprehension (p = 0.166) between the mild and moderate groups (Table 2, Fig. 1).

**Table 2:** Characteristics of the clusters.

	Cluster1	Cluster2	Cluster3	*P* value
Level of linguistic deficit	Severe	Moderate	Mild	
Number of participants	109	71	128	
WAB-AQ scores, mean ± SD (range)	13.100 ± 12.001 (0–44.6)	42.271 ± 16.918 (15.4–74.2)	72.017 ± 16.819 (26.3–93.7)	
Time post-onset, months, mean ± SD	6.798 ± 9.746	8.585 ± 9.899	7.408 ± 9.398	0.477^a^
Defined range of the WAB-AQ cutoff scores	0–30	30.1–50.3	50.4–93.7	
Group number of participants	123	55	130	
Group mean WAB-AQ score	13.058 ± 9.769	41.255 ± 5.816	75.235 ± 11.585	< 0.001^a^

*SD* standard deviation, *WAB-AQ* aphasia quotient of Western Aphasia Battery.

^a^1-way analysis of variance.

### Severe

Participants characterized as severe had poor verbal expression ability. Although some comprehension and repetition abilities are retained to some extent, it is still unlikely to participate in daily communication.

### Moderate

Participants characterized as moderate had good auditory comprehension ability, relatively. However, due to the impairment of language expression ability, it is partially difficult to participate in daily communication independently.

### Mild

Participants characterized as mild had retained relatively complete expression ability and auditory comprehension ability, who can participate in some daily communication activities independently.

## Discussion

The original version of WAB was designed based on the BDAE, accompanying certain system continuity^10^. Subsequently, various versions of WAB appeared in the study for the population with the linguistic deficit in different countries, languages, and diseases^18–20^. The severity of initial aphasia is closely related to the prognosis, which has been confirmed long ago^21^. Considering the above situation, we plan to conduct a secondary cluster analysis on those with WAB-AQ scores less than 93.8 based on the original score division of WAB, to further optimize the evaluation system of aphasia and facilitate the clinical application of aphasia evaluation.

In this study, we performed a division of linguistic deficit levels using cutoff scores. The benefit of using a cluster analysis to define impairment levels of the WAB-AQ assessment is that the cutoff points are derived using an objective quantitative method. In practical research, many scholars take WAB as an index to judge the type and severity of aphasia and evaluate the curative effect and outcome^22,23^. The existing stratification of aphasia may be more based on the subjective impression of clinicians. As described in the BDAE, 0 indicates that the patient has no meaningful language or auditory comprehension ability and 5 indicates that there is almost no discernible language impairment^24^. Although in the clinical application of nervous system diseases, the diagnosis of language defects needs to be combined with more clinical data, such as imaging, biochemistry, and pathophysiology, we hope to provide some convenience for language rehabilitation practitioners and patients through our research results.

In terms of the characteristics of linguistic deficit within each cluster, from the severe to the mild linguistic deficit group, there was an increase in WAB-AQ scores. In the WAB-AQ subtests, the scores of spontaneous speech, repetition, and naming increased with the decrease of the severity of aphasia, while there was no significant statistical difference in auditory comprehension between the mild and moderate groups. According to the scores of all subitems, we tried to describe the characteristics of the groups at three linguistic deficit levels from three aspects: language output, language reception, and communication, as shown in the research results. What we need to explain here is that the above characteristics are common features in the cluster and cannot represent the language characteristics of each individual in the cluster. Similar results were also obtained in this study: the spontaneous speech was correlated with naming (r = 0.862), which had the greatest impact on the WAB-AQ score (r = 0.947, 0.929).

The current study included individuals with post-stroke aphasia at various stages of the disease, which increases the heterogeneity of research objects. However, the results of univariate ANOVA showed that there was no significant difference in the mean TPO across groups.

In the future study, we can include language disorders caused by other diseases, such as primary progressive aphasia. There is no correlation analysis of aphasia types in this study, which can also be considered as a direction for future research.

## Conclusions

For PSA, the systematic cluster analysis of the WAB-AQ revealed 1 set of classification schemes (severe, moderate, mild).

## Data Availability

The datasets generated during the current study are not publicly available due to privacy or ethical restrictions but are available from the corresponding author on reasonable request.
