# Author Correction: Determining levels of linguistic deficit by applying cluster analysis to the aphasia quotient of Western Aphasia Battery in post-stroke aphasia

**DOI:** 10.1038/s41598-022-25024-5

**Published:** 2022-12-01

**Authors:** Zhijie Yan, Dongshuai Wei, Shuo Xu, Jingna Zhang, Chunsheng Yang, Xinyuan He, Chong Li, Yongli Zhang, Mengye Chen, Xiaofang Li, Jie Jia

**Affiliations:** 1 Department of Rehabilitation Medicine, The Third Affiliated Hospital of Xinxiang Medical University, The East Section of Hualan Avenue, Hongqi District, Xinxiang, 453000 China; 2 Department of Rehabilitation Medicine, Huashan Hospital, Fudan University, 12 Middle Wulumuqi Road, Jing’an District, Shanghai, 200040 China; 3 National Clinical Research Center for Aging and Medicine, Huashan Hospital, Fudan University, Shanghai, 200040 China

Correction to: *Scientific Reports* 10.1038/s41598-022-17997-0, published on 06 September 2022

The original version of this Article contained an error in the spelling of the author Jie Jia which was incorrectly given as Jia Jie.

The original Article has been corrected.

